# External validation of the rCAST for patients after in-hospital cardiac arrest: a multicenter retrospective observational study

**DOI:** 10.1038/s41598-024-54851-x

**Published:** 2024-02-21

**Authors:** Junki Ishii, Mitsuaki Nishikimi, Kazuya Kikutani, Michihito Kyo, Shingo Ohki, Kohei Ota, Mitsuhiro Fujino, Masaaki Sakuraya, Shinichiro Ohshimo, Nobuaki Shime

**Affiliations:** 1 Department of Emergency and Critical Care Medicine, Graduate School of Biomedical and Health Sciences, Hiroshima University, 1-2-3 Kasumi, Minami-ku, Hiroshima, 734-8551 Japan; 2 Department of Emergency and Critical Care Medicine, Nagoya University Graduate School of Medicine, 65 Tsurumi-cho, Showa-ku, Nagoya, 466-8550 Japan; 3 Department of Critical Care and Emergency Medicine, Otsu City Hospital, 2-9-9 Motomiya, Otsu, 520-0804 Japan; 4 Department of Emergency and Intensive Care Medicine, JA Hiroshima General Hospital, 1-3-3 Jigozen, Hatsukaichi, Hiroshima 738-8503 Japan

**Keywords:** Post-cardiac arrest syndrome, Neurological prognosis, In-hospital cardiac arrest, Risk classification, rCAST, Cardiology, Neurology, Risk factors

## Abstract

No established predictive or risk classification tool exists for the neurological outcomes of post-cardiac arrest syndrome (PCAS) in patients with in-hospital cardiac arrest (IHCA). This study aimed to investigate whether the revised post-cardiac arrest syndrome for therapeutic hypothermia score (rCAST), which was developed to estimate the prognosis of PCAS patients with out-of-hospital cardiac arrest (OHCA), was applicable to patients with IHCA. A retrospective, multicenter observational study of 140 consecutive adult IHCA patients admitted to three intensive care units. The area under the receiver operating characteristic curves (AUCs) of the rCAST for poor neurological outcome and mortality at 30 days were 0.88 (0.82–0.93) and 0.83 (0.76–0.89), respectively. The sensitivity and specificity of the risk classification according to rCAST for poor neurological outcomes were 0.90 (0.83–0.96) and 0.67 (0.55–0.79) for the low, 0.63 (0.54–0.74) and 0.67 (0.55–0.79) for the moderate, and 0.27 (0.17–0.37) and 1.00 (1.00–1.00) for the high-severity grades. All 22 patients classified with a high-severity grade showed poor neurological outcomes. The rCAST showed excellent predictive accuracy for neurological prognosis in patients with PCAS after IHCA. The rCAST may be useful as a risk classification tool for PCAS after IHCA.

## Introduction

Although the outcomes of in-hospital cardiac arrest (IHCA) patients have improved^1,2^, survival rates are still low, varying from 9 to 28%^3–7^. Since IHCA patients have received relatively little attention compared with out-of-hospital cardiac arrest (OHCA) patients, it is important to facilitate research focusing on this population that may improve their outcomes. One of the biggest issues is the lack of a universally established risk classification for patients with IHCA, despite the heterogeneity of this population. The precise estimation of severity among patients with IHCA can help with statistical comparisons in epidemiological studies, as well as in decision-making regarding the appropriate management strategy in clinical settings. Furthermore, risk classification can objectively guide healthcare providers and family members in joint decision-making about future treatment options for patients^8^.

The clinical characteristics of patients with IHCA differ substantially from those of patients with OHCA. For instance, among patients with IHCA, cardiac arrests are more likely to be witnessed, with respiratory causes among the most common aetiologies^3^. Additionally, the timing until recognition of cardiac arrest is typically shorter, and basic life support is initiated earlier^3^. Considering these potential differences, a risk classification tool focusing on post-cardiac arrest syndrome (PCAS) in patients with IHCA specifically may be needed. However, fewer risk classification tools for IHCA have been reported compared with OHCA^9,10^.

We previously validated the revised post-Cardiac Arrest Syndrome for Therapeutic hypothermia score (rCAST) as a risk classification tool for PCAS patients with OHCA^11,12^. The area under the receiver operating characteristic curve (AUC) of the rCAST for poor neurological outcome at 30 days of PCAS in patients with OHCA was approximately 0.9, which was regarded as excellent accuracy^13^. Five clinical variables for the calculation of the rCAST (initial rhythm, witness/time until return of spontaneous circulation [ROSC], pH, lactate, motor scale of Glasgow Coma Scale [GCS M]) can be easily measured even for patients with IHCA. We hypothesized that the rCAST could be also useful for estimating the severity and predicting neurological prognosis in patients with IHCA, similar to those with OHCA. This study aimed to investigate the applicability of the rCAST and the three severity grades of it in patients with PCAS after IHCA.

## Materials and methods

The study was carried out in accordance with the Declaration of Helsinki and the institutional review board of Hiroshima University approved this study, which waived the requirement for informed patient consent to ensure participant anonymity as stipulated in the Japanese government guidelines (Approval No. E2022-0054).

### Study design

This retrospective, multicenter observational study included 140 consecutive adult patients with post-cardiac arrest syndrome (PCAS) with IHCA who were admitted to three intensive care units (ICUs) (Hiroshima University Hospital, Japan Agricultural Cooperatives (JA) Hiroshima General Hospital, and Otsu City Hospital) between January 2015 and June 2022. The ROSC was defined as the spontaneous maintenance of circulation for 20 consecutive minutes. Hiroshima University Hospital is an academic quaternary care hospital with 742 beds, including 22 ICU beds; JA Hiroshima General Hospital is a tertiary care hospital with 531 beds, including eight ICU beds; and Otsu City Hospital is a tertiary care hospital with 401 beds, including eight ICU beds. The protocol for targeted temperature management (TTM), including the core temperature setting, depended on the protocol followed at each participating hospital. TTM is considered for cardiac arrest patients who are in coma (Glasgow Coma Scale [GCS] ≤ 8) after ROSC, according to the recommendation of the Japanese resuscitation guideline^14^.

Patients were excluded if they were under 18 years of age, had not lived independently prior to experiencing cardiac arrest (cerebral performance category [CPC] ≥ 3), or were treated with extracorporeal cardiopulmonary resuscitation (ECPR). In addition, patients with missing values for the variables that were required to calculate the rCAST were excluded.

### Dataset

Data were retrospectively collected from electronic chart reviews, including clinical histories (age, sex, CPC before cardiac arrest, etiology of cardiac arrest, situation surrounding the cardiac arrest, and past medical history), initial rhythm, laboratory data from venous blood gas, arterial blood gas, serum laboratory tests, and clinical courses before and after ROSC. The rCAST score points were calculated using clinical variables measured at the closest time from the cardiac arrest (no more than 6 h) (Supplementary Fig. S1).

### Outcome measurement

The primary outcome was neurological outcome at 30 days. We classified the neurological outcomes according to the results of evaluation of the CPC at 30 days: CPC 1, full recovery; CPC 2, moderate disability; CPC 3, severe disability; CPC 4, coma or vegetative state; CPC 5, death. CPC 1–2 were considered good neurological outcomes, while CPC 3–5 were considered poor neurological outcome^15^. The certified ICU specialists of the study team evaluated the CPC at 30 days before calculating the rCAST and analysis in a blinded manner. CPC at 30 days was determined according to the definition described in a previous study^15^ through retrospective electronic chart reviews. We referred to the charts written by physicians, nurses, or rehabilitation therapists. For example, patients who were reported to be independent in daily living activities were categorized into CPC ≤ 2, whereas those reliant on others were categorized into CPC of 3 or 4. The secondary outcome was mortality at 30 days.

### Statistical analysis

A complete case analysis was conducted in this study. Continuous variables were expressed as median (interquartile range 25–75), and categorical variables were expressed as proportions (%). The performance of the rCAST was assessed using discrimination and calibration. For discrimination, a receiver operating characteristic (ROC) curve was plotted, and the AUC was calculated. The discrimination of AUC of more than 0.9 was defined as outstanding, 0.8–0.9 as excellent, 0.7–0.8 as acceptable, 0.5–0.7 as poor, and 0.5 as no discrimination, based on a previous study^13^. For calibration, the Hosmer–Lemeshow test was performed. Calibration was also assessed by plotting the agreement between the observed outcomes and predicted probabilities^16,17^. Additionally, as a subgroup analysis, we plotted ROC curves and calculated the AUCs of rCAST for patients who underwent TTM and those who did not undergo TTM.

To compare the AUC of rCAST to those of the other two existing prognostic scores (Good Outcome Following Attempted Resuscitation [GO-FAR] score^18^ and Out-of-Hospital Cardiac Arrest [OHCA] score^10^), DeLong’s test was performed. Only 120 patients were analyzed for comparison with the OHCA score, as all 20 patients had missing values of no flow time for the calculation of the OHCA score.

Sensitivity and specificity were also calculated for the three severity grades of rCAST developed in our previous study (rCAST ≤ 5.5 was categorized as low severity, rCAST of 6.0–14.0 was moderate severity, and rCAST ≥ 14.5 was high severity)^11^. In the current study, specificity was defined as the proportion of patients with poor outcomes who were correctly identified, and the bootstrap approach (2000 times) was used to calculate the 95% confidence intervals (95% CI) for sensitivity and specificity. In addition, we investigated the thresholds for the rCAST for poor neurological outcomes in the analyzed patients, corresponding to 95% sensitivity, 95% specificity, and the maximum value of the Youden index^19^.

All reported *P* values were two-sided, and statistical significance was set at *P* < 0.05. All analyses were performed using R, version 4.1.3 (Vienna University of Economics and Business, Vienna, Austria) and JMP Pro 15 software (SAS Institute, Cary, NC, USA).

## Results

**Figure 1:**
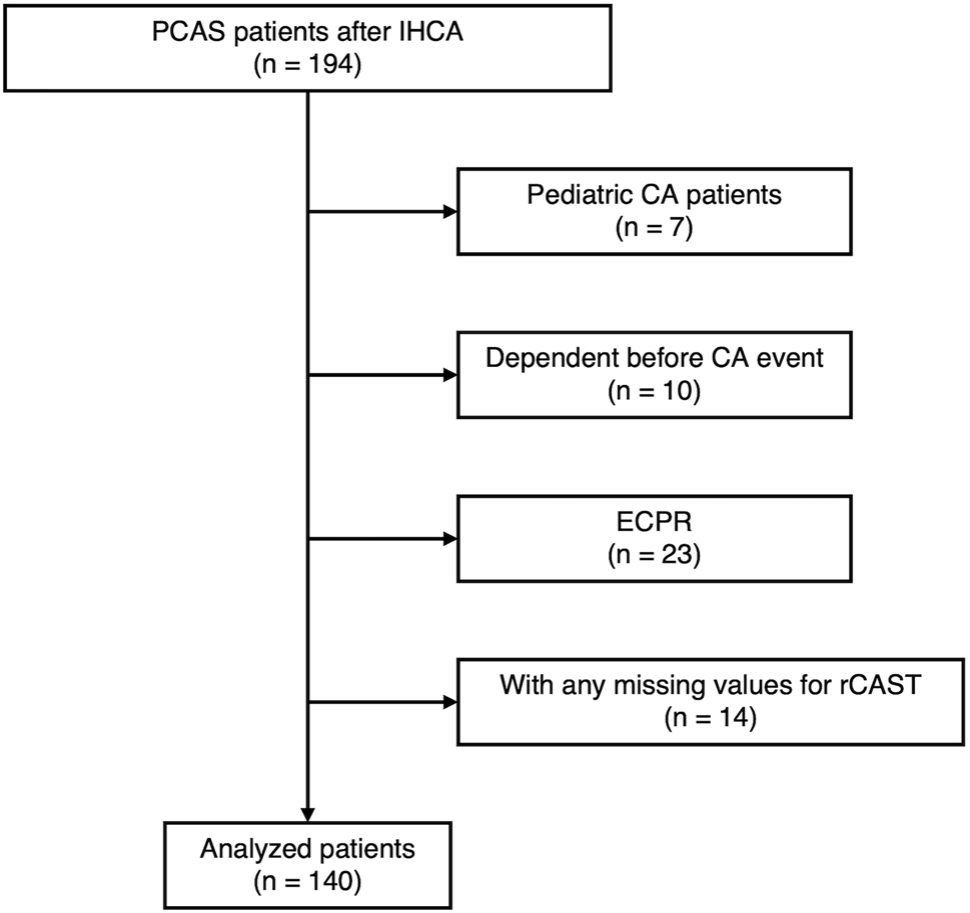
Flow chart of patient enrollment in this study. *PCAS* post-cardiac arrest syndrome, *IHCA* in-hospital cardiac arrest, *CA* cardiac arrest, *ECPR* extracorporeal cardiopulmonary resuscitation, *rCAST* revised post-cardiac arrest syndrome for therapeutic hypothermia score.

A patient flowchart for this study is shown in Fig. 1. Among 194 patients eligible for this study, 54 were excluded according to the preset criteria because they were under 18 years old (n = 7), were not independent before experiencing an IHCA (n = 10), received ECPR (n = 23), or had missing values for calculation of rCAST (n = 14).

**Figure 2:**
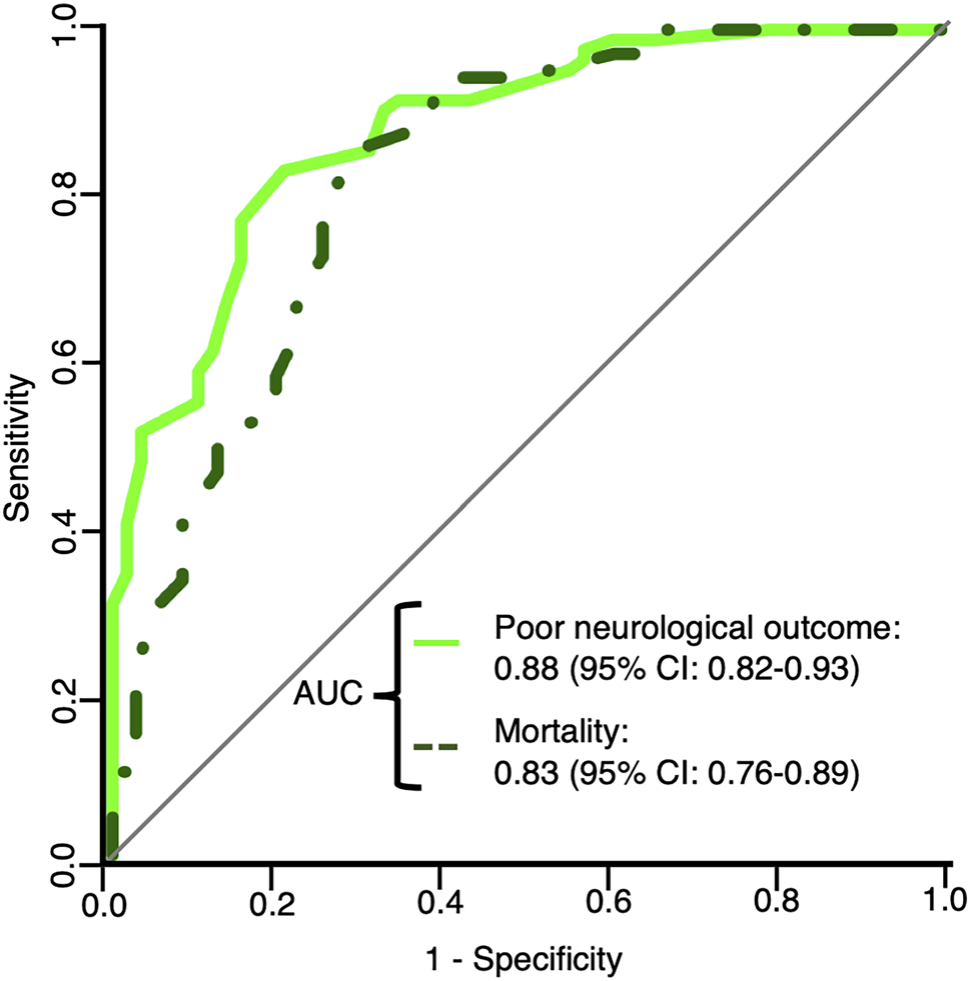
Receiver operating characteristic (ROC) curve of rCAST. The bold line indicates the ROC curve of rCAST for poor neurological outcomes at 30 days, whereas the dashed line indicates mortality at 30 days. *AUC* area under the ROC curve, *CI* confidence interval, *rCAST* revised post-cardiac arrest syndrome for therapeutic hypothermia score.

**Figure 3:**
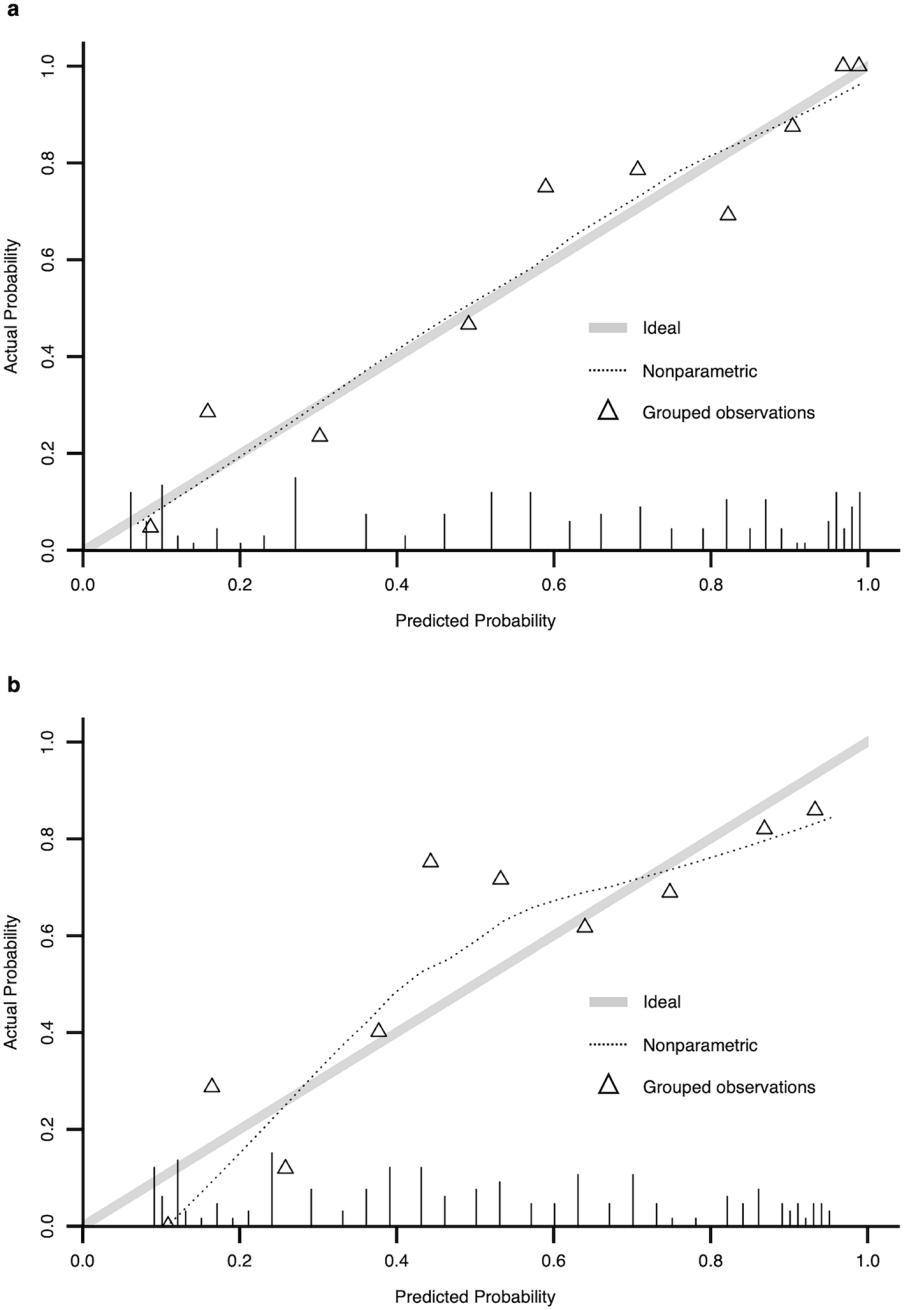
Calibration plots of rCAST. Calibration plots of the rCAST for poor neurological outcomes (**a**) and mortality (**b**) after 30 days. The dashed curve expresses a non-parametric estimate of the calibration relationship between the actual and predicted values, whereas the gray line expresses the ideal relationship (intercept of zero and slope of one). *rCAST* revised post-cardiac arrest syndrome for therapeutic hypothermia score.

**Figure 4:**
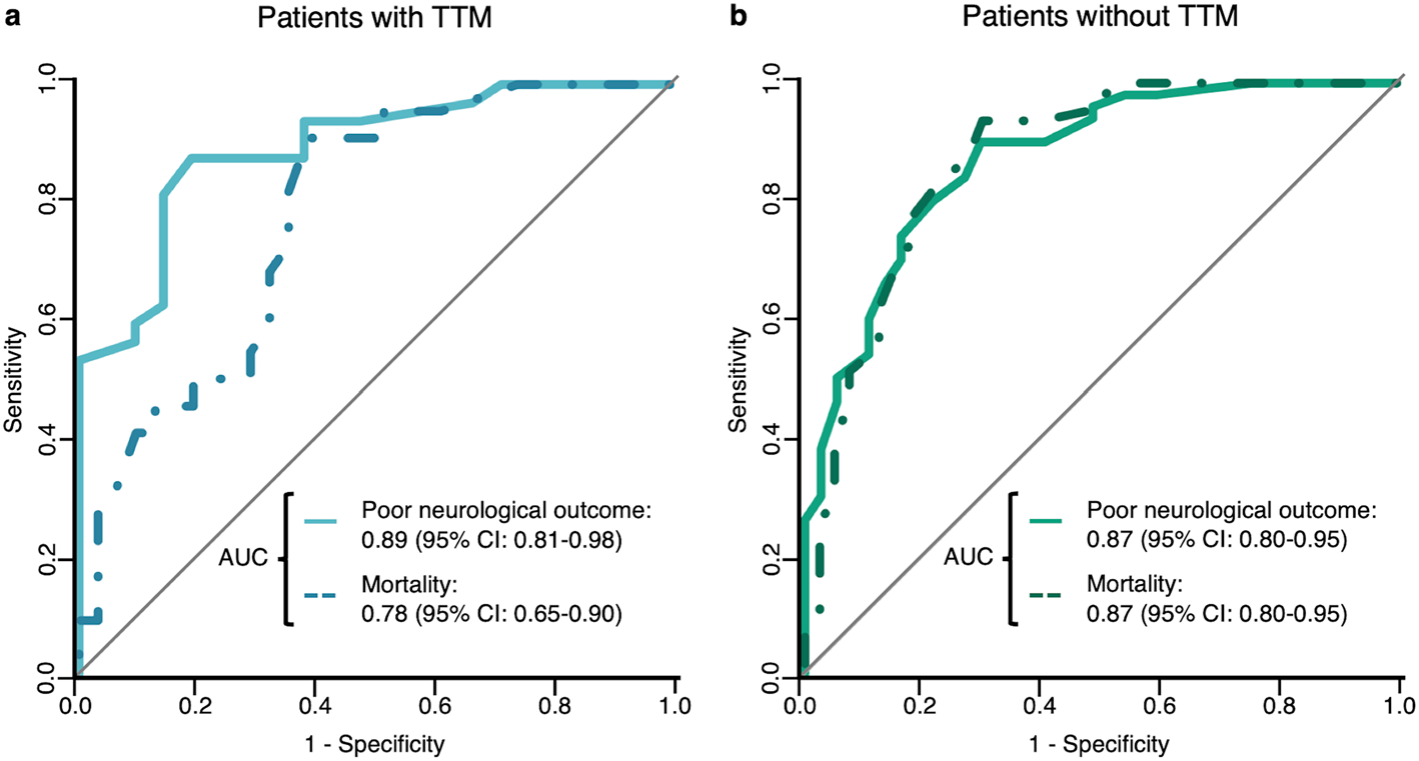
Receiver operating characteristic curves of rCAST for the patients with and without target temperature management. (**a**) The ROCs of rCAST for the patients who underwent TTM. (**b**) The ROCs of rCAST for the patients who did not undergo TTM. The bold line indicates ROC curve of rCAST for poor neurological outcome at 30 days, while the dashed line indicates mortality at 30 days. *TTM* target temperature management, *AUC* area under the ROC curve, *CI* confidence interval, *rCAST* revised post-cardiac arrest syndrome for therapeutic hypothermia score.

The baseline characteristics of the 140 patients are summarized in Table 1. Eighty-two patients (59%) had poor neurological outcomes at 30 days, while 69 patients (49%) died at 30 days. The AUCs of the rCAST for poor neurological outcome and mortality at 30 days were 0.88 (0.82–0.93) and 0.83 (0.76–0.89), respectively (Fig. 2). Calibration plots for poor neurological outcome and mortality at 30 days are shown in Fig. 3 (Hosmer–Lemeshow test: *P* = 0.705, poor neurological outcome; *P* = 0.099, mortality). Additionally, we performed a subgroup analysis of patients who underwent TTM and those who did not (Fig. 4). The AUCs for poor neurological outcome and mortality at 30 days in patients treated with TTM were 0.89 (0.81–0.98) and 0.78 (0.65–0.90), respectively, while those in patients without TTM were 0.87 (0.80–0.95) and 0.87 (0.80–0.95), respectively. The cutoff values for poor neurological outcomes among the analyzed patients corresponding to 95% sensitivity, 95% specificity, and the maximum value of Youden index and each sensitivity and specificity were 4.0 (sensitivity = 0.95, specificity = 0.45), 10.5 (sensitivity = 0.51, specificity = 0.97) and 7.0 (sensitivity = 0.83, specificity = 0.79), respectively.

**Table 1:** Patient characteristics.

	Overall (n = 140)	Good (n = 58)	Poor (n = 82)	*P* value
Age, years	71 (62‒78)	72 (58‒77)	71 (64‒79)	0.393
Sex, male, n (%)	92 (66)	38 (66)	54 (66)	> 0.999
Comorbidities				
Metastatic or hematologic cancer, n (%)	25 (18)	9 (16)	16 (20)	0.656
Bacteremia^a^, n (%)	6 (4)	3 (5)	3 (4)	0.692
Hepatic insufficiency^b^, n (%)	29 (21)	8 (14)	21 (26)	0.096
Renal insufficiency^c^, n (%)	49 (35)	18 (31)	31 (38)	0.474
Hypotension and hypoperfusion^d^, n (%)	48 (34)	17 (29)	31 (38)	0.367
Respiratory insufficiency^e^, n (%)	49 (35)	16 (28)	33 (40)	0.151
Etiology of cardiac arrest, cardiac, n (%)	41 (29)	27 (47)	14 (17)	< 0.001
Witness, n (%)	119 (85)	53 (91)	66 (80)	0.094
Bystander, n (%)	109 (78)	47 (81)	62 (76)	0.537
Low flow time^f^, min	9 (4–16)	6 (2–9)	13 (8–19)	< 0.001
Initial rhythm, shockable, n (%)	25 (18)	18 (31)	7 (9)	0.001
Adrenaline use until ROSC, n (%)	105 (75)	35 (60)	70 (85)	0.001
Blood examination at ROSC				
pH	7.13 (6.98‒7.28)	7.26 (7.12‒7.36)	7.03 (6.95‒7.16)	< 0.001
Lactate, mmol/L	7.9 (4.7‒12.6)	5.7 (3.2‒8.9)	10.2 (6.7‒14.0)	< 0.001
Cre, mg/dL	1.6 (1.0‒3.4)	1.3 (0.9‒3.7)	1.8 (1.2‒3.1)	0.125
Treatment				
PCI, n (%)	6 (4)	5 (9)	1 (1)	0.082
TTM, n (%)	53 (38)	21 (36)	32 (39)	0.860
Mortality at 30 days, n (%)	69 (49)	0 (0)	69 (84)	< 0.001

Data are presented as median and interquartile ranges (25th–75th percentile) or as absolute frequencies with percentages.

*ROSC* return of spontaneous circulation, *Cre* creatinine, *PCI* percutaneous coronary intervention, *TTM* targeted temperature management.

^a^Documented bloodstream infection in which antibiotic therapy has not yet been initiated or is still ongoing.

^b^Evidence of hepatic insufficiency within 24 h of the event, defined as total bilirubin > 2 mg/dL and aspartate transaminase (AST) > 2 times the upper limit of normal or cirrhosis.

^c^Requiring ongoing dialysis or extracorporeal filtration therapies or serum creatinine > 2 mg/dL within 24 h of the event.

^d^Any evidence of hypotension within 4 h of the event, defined as any of the following: systolic blood pressure < 90 mmHg or mean blood pressure < 60 mmHg; vasopressor or inotropic requirement after volume expansion; or intra-aortic balloon pump.

^e^Evidence of acute or chronic respiratory insufficiency within 4 h of the event, defined as any of the following: partial pressure of arterial oxygen/inspired oxygen fraction (PaO2/FiO2) ratio < 300, PaO2 < 60 mmHg, or arterial oxygen saturation (SaO2) < 90%; partial pressure of arterial carbon dioxide (PaCO2), end tidal carbon dioxide (ETCO2), or transcutaneous carbon dioxide (TcCO2) > 50 mmHg; spontaneous respiratory rate > 40 breaths/min or < 5 breaths/min; requirement for noninvasive ventilation or negative pressure ventilation; or requirement for ventilation via an invasive airway.

^f^Time from the initiation of cardiopulmonary resuscitation to ROSC.

The AUCs of rCAST for poor neurological outcome and mortality at 30 days were significantly higher than those of GO-FAR and OHCA scores, except for the OHCA score for mortality at 30 days (vs. GO-FAR score: 0.88 [0.82–0.93] vs. 0.67 [0.58–0.76], *P* < 0.001 for poor neurological outcome; and 0.83 [0.76–0.89] vs. 0.68 [0.60–0.77], *P* = 0.013 for mortality) (vs. OHCA score: 0.89 [0.83–0.94] vs. 0.81 [0.73–0.89], *P* = 0.031 for poor neurological outcome; and 0.84 [0.77–0.91] vs. 0.80 [0.72–0.88], *P* = 0.267 for mortality) (Supplementary Fig. S2).

Furthermore, we validated three previously established severity grades based on rCAST. The proportions of poor neurological outcome at 30 days in the low (rCAST ≤ 5.5), moderate (rCAST 6.0–14.0), and high (rCAST ≥ 14.5) severity grades were 17% (n = 8), 73% (n = 52), and 100% (n = 22), respectively, while those of the mortality at 30 days were 9% (n = 4), 65% (n = 46), and 86% (n = 19), respectively. All 22 patients classified as having a high-severity grade showed poor neurological outcomes at 30 days. The sensitivity and specificity of each severity grade for poor neurological outcomes were (0.90 [0.83–0.96], 0.67 [0.55–0.79]) in the low-severity grade, (0.63 [0.54–0.74], 0.67 [0.55–0.79]) in the moderate-severity grade, and (0.27 [0.17–0.37], 1.00 [1.00–1.00]) in the high-severity grade, respectively. The sensitivity and specificity of the three grades for mortality were (0.94 [0.88–0.99], 0.61 [0.49–0.72]) in the low-severity grade, (0.67 [0.55–0.77], 0.65 [0.54–0.76]) in the moderate-severity grade, and (0.28 [0.17–0.38], 0.96 [0.90–1.00]) in the high-severity grade, respectively (Table 2).

**Table 2:** Diagnostic accuracy of the rCAST for poor neurological outcome and mortality at 30 days.

rCAST prognostic category	Specificity	Sensitivity
Poor neurological outcome at 30 days		
Low severity (≤ 5.5) (n = 47)	0.67 (0.55‒0.79)	0.90 (0.83‒0.96)
Moderate severity (6.0–14.0) (n = 71)	0.67 (0.55‒0.79)	0.63 (0.54‒0.74)
High severity (≥ 14.5) (n = 22)	1.00 (1.00‒1.00)	0.27 (0.17‒0.37)
Mortality at 30 days		
Low severity (≤ 5.5) (n = 47)	0.61 (0.49–0.72)	0.94 (0.88‒0.99)
Moderate severity (6.0–14.0) (n = 71)	0.65 (0.54‒0.76)	0.67 (0.55‒0.77)
High severity (≥ 14.5) (n = 22)	0.96 (0.90‒1.00)	0.28 (0.17‒0.38)

Data are presented as mean (95% CI).

*rCAST* revised post-cardiac arrest syndrome for therapeutic hypothermia, *CI* confidence interval.

## Discussion

In this study, we evaluated the predictive accuracy of the rCAST for poor neurological outcomes and mortality at 30 days in PCAS patients with IHCA. Both AUCs of rCAST were regarded as having excellent predictive accuracy, based on previous study results^13^, which was comparable to previous results conducted in OHCA.

Notably, the false-positive rate was 0.0% (0/22) for patients categorized into the high-severity grade based on rCAST, indicating that patients in the high-severity grade may have a zero probability of a good neurological outcome at 30 days. However, it should be noted that the purpose of the scoring system is not to directly influence withdrawal of life-sustaining therapy decisions. Instead, predictions on the precise neurological prognosis of patients should also be made by more decisive examinations, such as electroencephalography or somatosensory evoked potential testing, performed several days after ICU admission^20^. Nevertheless, we believe that risk classification is helpful in guiding healthcare providers and family members in joint decision-making regarding future treatment options of patients.

Recent studies on OHCA patients suggest a possibility that the treatment effect size of TTM at the lower setting temperature may be different based on their severity^21,22^. Moreover, our previous study showed that the treatment effect size was different by score points on the rCAST and that the patient group that is particularly likely to derive benefit from TTM at the lower setting temperature is the group with PCAS of moderate-severity grade on rCAST^23^. Although further research is needed, this may also be applicable to IHCA patients, indicating that the risk classification with the rCAST before TTM may be helpful in identifying IHCA patients who benefit most from TTM at the lower setting temperature^24^.

We would also like to emphasize the advantages of using the rCAST in clinical settings compared with using other predictive scores. As all five variables of the rCAST are easily and routinely obtained in clinical practice, we believe that the number of patients for whom the rCAST cannot be calculated is lower in actual practice. The percentage of patients for whom the rCAST was unavailable due to missing values in our study was only 9.1% (14/154). Given the simple rCAST formula, we believe that it may be more practical in clinical settings compared with other predictive scores for PCAS with IHCA^10,18,25^.

Additionally, considering that the rCAST was originally developed for PCAS patients who were going to be treated with TTM^11^, it is not surprising that we observed a trend of slightly higher predictive accuracy of the rCAST for patients treated with TTM than for those without TTM. Nevertheless, the predictive accuracy of the rCAST was still excellent in patients without TTM, suggesting that it is useful regardless of TTM.

This study had several limitations. First, it was based on retrospective data, although its validity can be generalized with data enrollment from multiple medical sites. Further prospective studies with larger sample sizes are hence warranted to exclude the effects of self-fulfilling prophecy bias. Second, we performed complete case analysis in this study. However, we believe that it did not influence the outcome greatly and the biases were minimal, because the proportion of excluded patients was only 9.1% (14/154). Third, the AUC of the GO-FAR score for poor neurological outcome in our study was lower than that of the original report (0.67 vs 0.78). This may be explained by the difference in the primary outcome, that is, because the GO-FAR score was originally developed to predict CPC 1^18^. Also, the GO-FAR score was developed for the purpose of pre-arrest prediction, and the variables were limited to parameters measurable only before cardiac arrest. It may be another reason for the lower AUC of GO-FAR score compared with those of rCAST, which includes parameters measurable after cardiac arrest. In addition, we did not compare the predictive accuracy of the rCAST with those of other prediction scores, such as the Cardiac Arrest Hospital Prognosis (CAHP)^26^ and MIRACLE_2_ scores^27^, which would warrant further research to pursue the better prediction model for IHCA. Also, our findings for the comparison of predictive accuracies between the rCAST and OHCA score should be evaluated prospectively because we excluded 20 patients owing to missing values for the OHCA score in the comparison, which can lead to selection bias. Finally, the primary outcome of our study was neurological outcome at 30 days. Few patients undergo any changes in the outcome between 30 and 90 days (from CPC ≤ 2 at 30 days to CPC > 3 at 90 days or from CPC > 3 at 30 days to CPC ≤ 2 at 90 days)^28^; therefore, the predictive accuracy of the rCAST for the long-term outcome of PCAS patients with IHCA may need further confirmation.

## Conclusions

The rCAST showed excellent predictive accuracy for neurological prognosis in patients with PCAS after IHCA. The rCAST may be useful as a risk classification tool for PCAS after IHCA.

### Supplementary Information

Supplementary Figures.

## Data Availability

The datasets used and analysed during the current study are available from the corresponding author on reasonable request.
