# Supplementary material for: External validation of the rCAST for patients after in-hospital cardiac arrest: a multicenter retrospective observational study
Source: Sci Rep. 2024 Feb 21;14:4284. doi: 10.1038/s41598-024-54851-x (PMC10882058; doi:10.1038/s41598-024-54851-x)
Supplement: Supplementary file 1 — Supplementary Figures. [file 41598_2024_54851_MOESM1_ESM.pdf]

Supplementary Fig. S1 Calculation formula and severity/risk classification of rCAST

a

	Variables	Score points 0	1	2	3
Variables	Initial Rhythm (x_1)	Shockable	Non Shockable		
	Witness / until ROSC time (x_2)	< 20 min	20min ≤	No Witness	
	pH (x_3)	≥ 7.31	7.30-7.16	7.15-7.01	7.00 ≥
	Lactate (mmol/L) (x_4)	≤ 5.0	5.1-10.0	10.1-14.0	14.1 ≤
	GCS M (x_5)	≥ 2	1		

b

rCAST = 1.0 * (x_1) + 2.0 * (x_2) + 2.5 * (x_3) + 0.5 * (x_4) + 4.5 * (x_5)

c

rCAST	Severity
≤ 5.5	Low
6.0-14.0	Moderate
≥ 14.5	High

(**a**) The rCAST can be calculated with five variables that are commonly evaluated during resuscitation and after ROSC. (**b**) Formula to calculate rCAST. (**c**) Severity grades according to the calculated rCAST. ROSC, return of spontaneous circulation; GCS M, motor scale of Glasgow Coma Scale; rCAST, revised post-Cardiac Arrest Syndrome for Therapeutic hypothermia score.

Supplementary Fig. S2 Comparison of receiver operating characteristic (ROC) curves between rCAST, and GO-FAR and OHCA scores

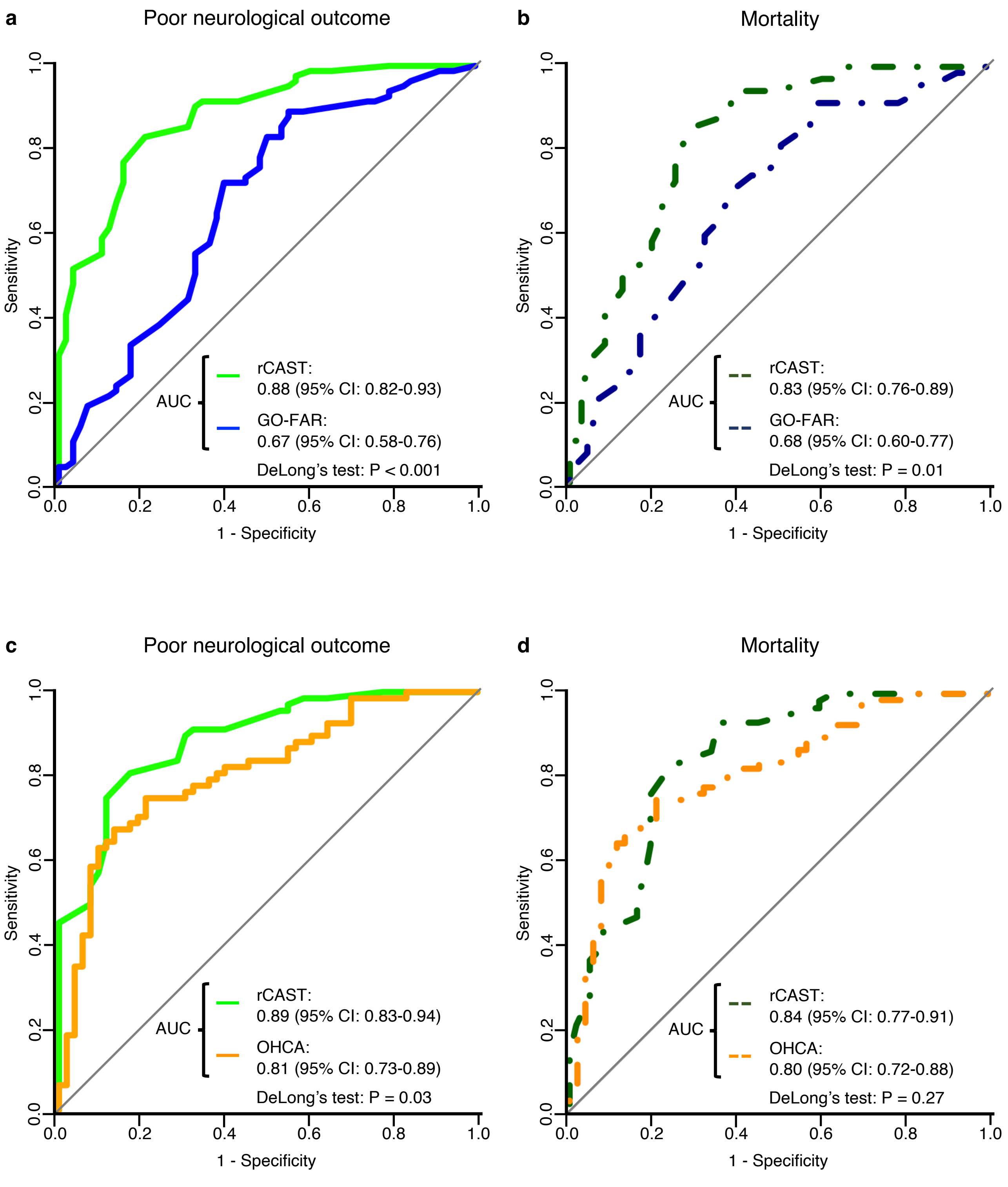

ROC curves of rCAST for poor neurological outcome at 30 days and mortality at 30 days were compared with those of GO-FAR scores (**a** and **b**) and OHCA scores (**c** and **d**), respectively. The DeLong's test was performed to compare the area under the ROC curve of these scores. AUC, area under the ROC curve; rCAST, revised post-Cardiac Arrest Syndrome for Therapeutic hypothermia score; CI, confidence interval; GO-FAR, Good Outcome Following Attempted Resuscitation score; OHCA, Out-of-Hospital Cardiac Arrest score.
